# Energy Expenditure, Carbohydrate Oxidation and Appetitive Responses to Sucrose or Sucralose in Humans: A Pilot Study

**DOI:** 10.3390/nu11081782

**Published:** 2019-08-01

**Authors:** Christine Chern, Sze-Yen Tan

**Affiliations:** 1 School of Pharmacy and Medical sciences, University of South Australia, Adelaide, SA 5001, Australia; 2 Institute for Physical Activity and Nutrition (IPAN), School of Exercise and Nutrition Sciences, Deakin University, Geelong, VIC 3220, Australia

**Keywords:** non-nutritive sweeteners, carbohydrate, oxidation, appetite, food intake

## Abstract

Background: In light of obesity, replacing sugar with non-nutritive sweeteners is commonly used to reduce sugar content of food products. This study aimed to compare human energy expenditure (EE), carbohydrate oxidation and food intake after the ingestion of test foods sweetened with sucrose or a non-nutritive sweetener. Methods: This was an acute crossover feeding study that entailed consumption of three test foods: jelly sweetened with 50 g sucrose (SUCROSE), with 120 mg of sucralose only (NNS), or 120 mg sucralose but matched in carbohydrate with 50 g maltodextrin (MALT). On test days, participants arrived at the research facility after an overnight fast. Resting energy expenditure (indirect calorimeter) was measured for 30 min followed by jelly consumption. Participants’ EE and substrate oxidation were measured for 90 min subsequently. After EE assessment, participants completed a meal challenge before leaving the research facility, and recorded food intake for the remaining day. Subjective appetite ratings were assessed before and after test foods and meal challenge. Results: Eleven participants completed the study. EE was higher in SUCROSE and MALT than NNS, but not statistically significant. Carbohydrate oxidation was SUCROSE > MALT > NNS (*p* < 0.001). Earlier and bigger rise in carbohydrate oxidation was observed in SUCROSE than MALT, although both were carbohydrate-matched. NNS did not promote energy expenditure, carbohydrate oxidation or stimulate appetite. Conclusions: Foods sweetened with sucrose or non-nutritive sweeteners but matched in carbohydrate content have different effects on human EE and carbohydrate oxidation. Sucralose alone did not affect EE, but lower energy in the test food from sugar replacement was eventually fully compensated. Findings from this pilot study should be verified with bigger clinical studies in the future to establish clinical relevance.

## 1. Introduction

The global prevalence of overweight and obesity is increasing and it increases the risk of several chronic diseases such as type 2 diabetes and cardiovascular disease [1,2]. Together with its co-morbidities, overweight and obesity increases mortality rates and adds burden to the healthcare system.

An increase in energy intake is one of several factors responsible for the rising prevalence of overweight and obesity [3]. In recent years, epidemiological studies have linked obesity with dietary sugar intake [4,5], and concluded that reducing sugar intake reduces prevalence of overweight and obesity [6]. The World Health Organization recommends a reduction in free sugar intake to no more than 10% of total daily intake [7]. A number of countries such as Mexico, Brazil and France have successfully reduced the demand for sugar-sweetened beverages through the introduction of sugar tax, and this may drive the reduction of added sugar during food processing by the food industry [8].

The intake of natural sugars from major food groups such as fruits, vegetables and dairy foods should not be limited as they are good sources of nutrients such as vitamin A, vitamin C, vitamin D, potassium, calcium, etc. Therefore, a common strategy to reduce total sugar intake is to avoid consumption of high-sugary discretionary foods, or choose healthier alternatives where sugars are replaced with non-nutritive sweeteners (NNS).

Sugar alcohols (e.g., sorbitol, xylitol, maltitol, etc.) are a group of NNS that provide sweetness that is comparable to table sugars [9]. Although sugar alcohols contain energy, these sweeteners are not well-digested and absorbed by the body and; therefore, provide minimal energy when consumed [10]. Another category of NNS possesses high sweetness potency [9] and some examples from this category include acesulfame potassium, alitame, rebaudioside A, aspartame, cyclamate, neotame, saccharin and sucralose. Because of the high sweetness potency, a very small amount of NNS is required to match the sweetness levels provided by sugars (often in milligrams). Hence, these sweeteners provide little or no energy to the body. All NNS have different chemical structures and their metabolism and health implications have been extensively discussed in a previous review [11]. Of all NNS, sucralose has a chemical structure that has the closest resemblance to sucrose (C_12_H_22_O_11_), where three hydrogen atoms in sucrose are replaced with chlorine in sucralose (C_12_H_19_Cl_3_O_8_). Sucralose is pH and heat-stable, and it is widely used in food products that requires heat application [12,13].

Currently, eight NNS (i.e., advantame, aspartame, acesulfame-K, neotame, saccharin, sucralose, stevia and monk fruit extracts) are considered to be generally recognized as safe (GRAS) by the US Food and Drug Authority and available to consumers [14]. Unlike sugars, NNS do not increase blood glucose [15]. However, there are also concerns that NNS may stimulate appetite and food intake, and subsequently weight gain [16,17]. These concerns appear to be supported by findings from a number of epidemiological studies, where the consumption of NNS foods or beverages was associated with higher body weight [18,19]. However, scientists also pointed out that such relationship may be reverse-causation, where people with higher body weight tend to include diet products to assist in weight management. Therefore, a better understanding of how NNS may influence acute energy balance, namely energy expenditure and energy intake, is needed.

The primary aim of this study was to compare energy expenditure and carbohydrate oxidation patterns after the consumption of test foods sweetened with table sugar (SUCROSE), sucralose (NNS), or sucralose but matched SUCROSE in energy and carbohydrate content with the addition of bland carbohydrate maltodextrin (MALT). The secondary aim was to compare appetitive responses and total daily intake following these test foods.

## 2. Materials and Methods

### 2.1. Study Design

This was an acute randomised, crossover study that included one baseline visit and three test visits.

### 2.2. Experimental Protocol

**Figure 1:**
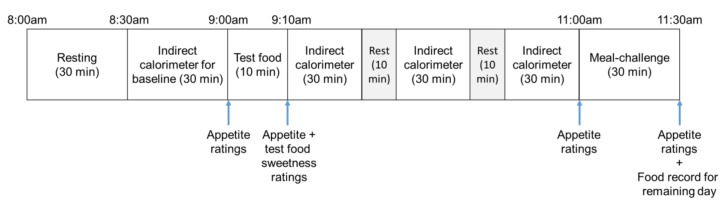
Experimental protocol on test day outlining study activities and time points when measurements were taken during the study.

At baseline, participants provided their written consent and had their height, body weight and eating behaviours assessed. Prior to each test day, the participants were reminded to avoid alcohol and strenuous exercise, consume their habitual dinner, and fast overnight for 10 h. On the test days, participants arrived between 08:00 and 09:00 am, voided urine, and had their body weight measured in a standing position with minimal clothing on. Following that, the participants rested for 30 min before their resting energy expenditure (REE) was assessed for 30 min. After REE, the participants consumed a test food within 10 min, followed by the measurements of postprandial gaseous exchanges (oxygen consumption and carbon dioxide production) for 90 min using the same indirect calorimeter, with a 10 min break every 30 min indirect calorimeter measurement, where the participants remained in a supine position without the hood. After the total 90 min calorimeter measurements, participants completed a meal challenge, where they were provided with 300 ml of plain water and bread with jam (sweet) and ham (savoury) toppings. Participants were asked to eat until they were comfortably full, which was defined as a fullness level where participants will not be eating other foods in the subsequent 3 to 4 h [20,21]. Appetite sensations were assessed pre- and post-consumption of test foods, as well as pre- and post-meal challenge, and sweetness levels of test foods were assessed immediately after ingestion of test foods. After the meal challenge, participants recorded food intake to determine their free-living energy intake for the rest of the day. The experimental protocol on test day is depicted in Figure 1. Test visits were repeated at least three days apart to normalize habitual food intake for males, and four weeks for females to ensure that experiment was performed at the same phase of the menstrual cycle, until all test foods were consumed. Participants were also asked to maintain their habitual dietary intake and physical activity level during the washout period. This study was conducted according to the guidelines laid down in the Declaration of Helsinki and all procedures involving human subjects were approved by the Human Research Ethics Committee (approval number #0000033058). Written informed consent was obtained from all participants, and they were able to withdraw from the study at any time without consequences. This trial has also been registered with the Australian and New Zealand Clinical Trial Registry (ACTRN12614000879662).

### 2.3. Study Participants

Participants were recruited from both the University and the local community. The inclusion criteria of this study were: (1) adults males (18–65 years old) or females (18–45 years old, pre-menopausal), (2) non-smokers, (3) normal or overweight (BMI 18.5–30.0 kgm^−2^), (4) weight changes of <5 kg in the past 3 months, (5) no diseases or not taking any medications that affect metabolism and appetite, and (6) no food allergies and have consumed diet food products containing NNS before.

### 2.4. Study Test Foods

This study included three types of jelly made with 100 g low-fat milk (274 kJ, 5.8 g carbohydrate, 7.7 g protein, 1.2 g fat) and 4.7 g gelling powder (carrageenan, brand: Jel-it-in, Queen Fine Foods Pty. Ltd., Alderley, QLD, Australia), sweetened with: (1) SUCROSE: 50 g sucrose (sweet carbohydrate), (2) NNS: 120 mg of sucralose only (sweet, no carbohydrate), and (3) MALT: 120 mg sucralose and matched 50 g carbohydrate as bland maltodextrin (Poly-Joule, Nutricia) (sweet and carbohydrate). The mixture was heated to dissolve all ingredients. The amount of sucralose used in MALT and NNS provided equal level of sweetness compared to SUCROSE, as determined through a sensory evaluation prior to this experiment.

Both SUCROSE and NNS test foods were equally sweet, but they differed in energy and carbohydrate content and may; therefore, have differential effects on human energy expenditure and carbohydrate oxidation in this study [22]. For this reason, a third test food, MALT, was included and its sweet taste came from sucralose but it has same energy and carbohydrate content (from maltodextrin) as the SUCROSE test food. Maltodextrin is a common ingredient used in food productions, is bland and hence did not alter the sweetness levels when added to the NNS test food containing 120 mg of sucralose. Therefore, SUCROSE and MALT were matched in sweetness levels, energy, and carbohydrate content to allow fair comparisons of metabolic and appetitive responses between types of sweeteners. NNS test food was chosen to examine the effects of non-nutritive sweetener alone. The order of test food consumption was randomised using an online research randomizer (www.randomizer.org) and participants were blinded to the type of test food they received at each test visit.

### 2.5. Measures

Anthropometry: Height was measured with a Seca stadiometer (to the nearest 1 mm) and body weight with a Tanita scale (Tanita BF-680, Tanita Inc., Japan, to the nearest 0.1 kg). Height and weight measurements were taken in duplicates and averaged. A third measurement was taken when height and weight measurements differed by more than 5 mm or 0.5 kg, respectively, and the average of the two closest measurements was taken. Eating behaviour was assessed at baseline using the validated three-factor eating questionnaire [23] to eliminate volunteers who may have unusual eating behaviour that could affect appetite ratings and food intake during meal challenge.

Energy expenditure and substrate oxidation (primary outcomes): Energy expenditure and substrate oxidation was assessed using a ventilated-hood indirect calorimeter (TrueMax 2400, Parvomedic Inc., Sandy, UT, USA). The machine was calibrated prior to each test visit using a standard 3.0 L gas syringe and standard gas (16.00% O_2_, 0.986% CO_2_ and balance nitrogen) to ensure accuracy of readings. Measurements were converted to standard temperature, pressure, and dry. Continuous gaseous exchanges were calculated as 10 min averages. For resting energy expenditure measurement, the final 20 min (stable state) of the 30 min measurement was used. Oxygen consumption and carbon dioxide production were used to calculate energy expenditure [24] and non-protein substrate oxidation rates [25] using previously published equations. 

Appetite and food intake (secondary outcomes): The appetite ratings of participants were assessed with validated 100-mm visual analogue scales (VAS) [26]. Sweetness level of test foods was also assessed using a horizontal 100-mm VAS anchored at “not sweet at all” on the left and “extremely sweet” on the right. A food diary was used to record free-living food intake for the remaining day. An explanatory page with instructions on how to document food intake accurately was provided with the diary, and food diaries were checked by researchers for completeness when they were returned. Dietary analysis was performed using the AUSNUT 2013 database in FoodWorks Professional (Xyris Software Pty. Ltd., Spring Hill, QLD, Australia).

### 2.6. Statistical Analysis

Descriptive statistics are presented as mean ± standard deviation. Postprandial EE and carbohydrate oxidation were calculated and presented as changes from baseline, as well as total over 90 min study period as area under the curve (AUC) using trapezoidal rules. The 90 min EE and carbohydrate oxidation was also adjusted for body weight taken on the test days, using regression residuals from a regression equation as following:

Y = a_0_ + a_1_ × Body Weight (kg),

where a_0_ is a constant and a_1_ is a coefficient.

The comparisons AUC and changes in EE, carbohydrate, appetite and sweetness ratings, and dietary intake following the consumption of three test foods were compared using general linear model for repeated measures ANOVA with Bonferroni corrections. All statistical analyses were performed with statistical package SPSS (version 23.0.0, IBM Corp, Armonk, NY, USA). As there was no previous study comparing the postprandial substrate oxidation effects induced by different sweeteners, sample size was guided by a study we previously conducted [27], where significant differences in substrate oxidation were detected with a study sample of 12 participants. Fifteen participants were targeted to account for attrition of 20%.

## 3. Results

Fifteen participants were recruited and 11 completed the study. Four withdrew from the study prior to the commencement of study due to difficulty in scheduling study visits. Participants who completed all test visits were eight males three females, aged = 24.9 ± 6.1 years, baseline body weight = 72.9 ± 19.0 kg (males = 81.0 ± 19.8 kg; females = 54.8 ± 8.4 kg), BMI = 25.0 ± 4.7 kgm^−2^ (males = 26.4 ± 5.5 kgm^−2^; females = 21.5 ± 3.1 kgm^−2^), and body fat = 20.3% ± 4.7% (males = 20.3% ± 8.7%; females = 22.3% ± 7.9%). Throughout all test visits, participants’ body weight did not change significantly from baseline: visit 1 = 0.0 ± 0.9 kg, visit 2 = −0.2 ± 0.8 kg, and visit 3 = 0.0 ± 0.7 kg. The jellies were rated as equally sweet by participants after consumption: SUCROSE = 62 ± 17 mm, NNS = 61 ± 13 mm, MALT = 64 ± 21 mm (repeated measures ANOVA, *p* = 0.911).

Table 1 summarises the unadjusted and adjusted (for body weight) values for total EE, carbohydrate and fat oxidation over the 90 min measurement period using an indirect calorimeter following test food was ingestion. The unadjusted variables were significantly different between all test foods (*p* < 0.05); the 90 min EE was almost statistically significant after adjustment for body weight (*p* = 0.050). The unadjusted EE were significantly higher in both SUCROSE and MALT than NNS. Carbohydrate oxidation rates were SUCROSE > MALT > NNS, and fat oxidation was significantly lower in SUCROSE than MALT and NNS.

**Figure 2:**
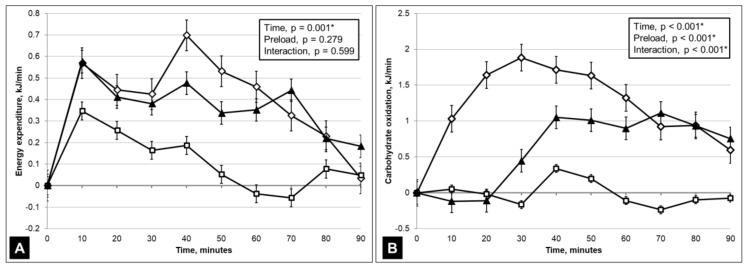
Temporal changes in energy expenditure (**A**) and carbohydrate oxidation (**B**) from resting values over 90 min following the ingestion of three sweet test foods. Values are presented as changes from baseline as means of *n* = 11 participants, and error bars represent standard errors (SE): Diamonds (◊)—SUCROSE (test food with 50g sucrose); triangles (▲)—MALT (test food with 120mg sucralose and 50g maltodextrin)squares (□)—NNS (test food with 120mg sucralose only);. * Statistically significant, general linear model for repeated measures ANOVA, *p* < 0.05.

As resting EE and carbohydrate oxidation rates were not significantly different between all study visits, data on these outcomes were also presented as patterns of changes from resting values after the ingestion of test foods in Figure 2. There were significant time effects on EE changes (*p* = 0.001) but they did not differ between test foods (F (8.3, 125.0) = 0.811, *p* = 0.599) (Figure 2A). However, significant interaction effects were found for carbohydrate oxidation (F (10.2, 153.5) = 6.117, *p* < 0.001) during the 90 min postprandial assessment (Figure 2B).

**Figure 3:**
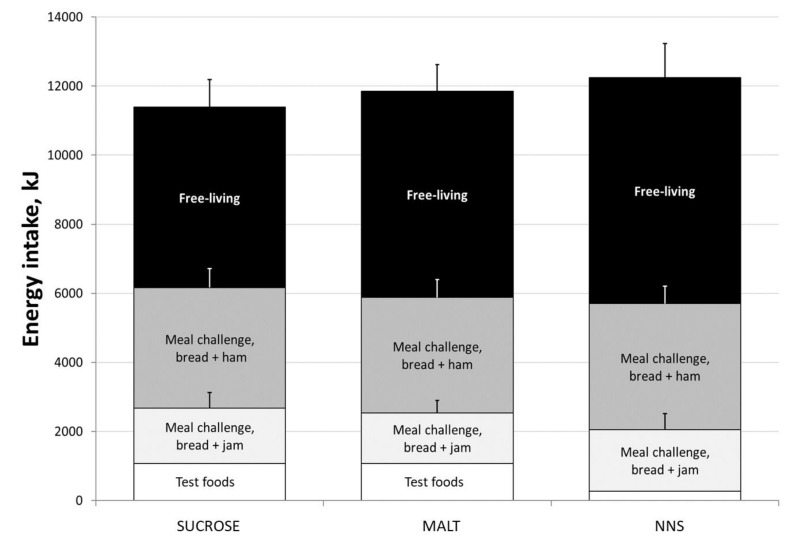
Total daily energy intake of participants from test foods, meal challenge, in a free-living environment on three test days. Bars represents total daily energy intake of study participants (*n* = 11) after each test food. Each bar is further divided into energy intake from the study test food, intake during meal challenge (bread with jam or ham toppings), and intake on the remaining day. Error bars represent standard error of measurement. Total daily, meal challenge (bread + ham and bread + jam), and free-living energy intake were not significantly different among the three test foods.

Appetite ratings pre- and post-test food ingestion, as well as pre- and post-meal challenge are presented in Table 2. No significant interaction effects of test foods on appetite ratings were found. Food intakes at meal challenge (bread with jam, *p* = 0.863; bread with ham, *p* = 0.874), in a free-living environment (*p* = 0.585), and total daily intake including test foods (*p* = 0.838) did not differ between test foods (Figure 3).

## 4. Discussion

This study was designed to examine how the re-formulation of a high-sugar food product with sucralose to reduce added sugar content may affect energy expenditure, carbohydrate oxidation, appetite and food intake. During the 90 min postprandial period, the EE was comparable between the SUCROSE and MALT test foods but both were higher than in NNS (Table 1 and Figure 2A). This observation was consistent with the evidence that postprandial EE is predominantly determined by the energy content of a meal [28].

Postprandial carbohydrate oxidation rate was significantly lower in NNS than SUCROSE and MALT (Table 1), which is likely to be explained by the higher carbohydrate content in SUCROSE and MALT. The temporal carbohydrate oxidation patterns of all jellies differ significantly (Figure 2B) although NNS did not alter carbohydrate oxidation. Interestingly, SUCROSE and MALT jellies produced different carbohydrate oxidation patterns although they have matched carbohydrate content. A higher and earlier carbohydrate oxidation peak was observed in SUCROSE, which subsequently converged with the MALT carbohydrate oxidation rates from 70 min onwards. As a result, the carbohydrate oxidation rate of SUCROSE was also significantly higher than MALT over 90 min.

There are two potential explanations for our novel finding on the differential carbohydrate oxidation patterns between SUCROSE and MALT. In the first scenario, maltodextrin is a polysaccharide and may be digested and absorbed slower than sugars (disaccharide) in SUCROSE test food, hence producing a lower and delayed carbohydrate oxidation. A potential counterargument is that maltodextrin is considered as a short-chain carbohydrates that is rapidly digested and release glucose for absorption [29]. In the second scenario, the differential carbohydrate oxidation patterns may be explained by sweet taste stimulation from two different types of sweeteners. Sweet taste perception stimulated by sugars has been shown to initiate carbohydrate-specific physiological responses such as salivary alpha-amylase content and hormones that are involved in blood glucose regulation such as insulin and GLP-1 [30,31,32]. Such effects were specific to sugars and were not seen when sweet taste was stimulated by NNS [15,30,33,34,35,36,37,38,39,40,41,42,43,44,45,46,47], which may be due to two distinctive pathways [48] and part of brain [49] of sweet taste activation. Previously, sensory stimulation has also been shown to alter energy expenditure [50,51]. Therefore, it is likely that sweet taste from sugars explained the earlier and greater increase in carbohydrate oxidation after SUCROSE test food. Carefully designed studies are needed to confirm this speculation.

There were indications from animal studies that NNS may stimulate appetite and food intake [10]. However, our study did not observe significant acute heightened appetite from the consumption of the NNS test food. However, it should be highlighted that energy gaps between NNS and SUCROSE and MALT were fully and slightly over-compensated for the remaining day. Our observations were consistent with a systematic review of observational and short-term interventional trials, where NNS use did not increase appetite, food intake or weight gain in humans [52]. In addition, preference for sweet foods was also not observed after jellies made with sucralose (MALT and NNS), as demonstrated by the comparable amount of bread with jam consumed during the meal challenge. 

This study has a number of strengths. The crossover design and carefully matched sweetness level of test foods allowed the participants to be tested in a blinded manner, hence minimise the cognitive compensation in food intake after test sessions. This study was also an early study to investigate how product formulation may have implications on energy balance via energy expenditure and energy intake. However, there were also limitations that restricted the interpretation of our data. First, this was a pilot study with small sample size to compare how sweetener types affect energy expenditure and carbohydrate oxidation in humans, which has not been studied before. Second, the digestion rates between sucrose and maltodextrin were not measured in this study and should; therefore, be considered in future studies. Future studies could measure blood glucose concurrently over 90 min to confirm if the digestion of SUCROSE and MALT and absorption of glucose may explain differential carbohydrate oxidation patterns observed in this study. Third, bread with jam is not a traditional lunch meal, and the cultural influence or expectation may have diluted the preference for sweet foods after sweet test foods. Fourth, the length of the study was not long enough to allow carbohydrate oxidation to return to baseline in order to examine if the total carbohydrate oxidized following SUCROSE and MALT are comparable. Fifth, although our study has sufficient statistical power to detect the differential carbohydrate oxidation rates between test foods, the relatively small sample size and young population in this exploratory study may not be representative of the general population. Sixth, this study assessed only one type of non-nutritive sweetener and it is unknown if findings can be extended to other non-nutritive sweeteners as they may have different chemical structures and sweet taste potency [9]. A recent randomized, controlled study showed that different non-nutritive sweeteners affected energy intake and have different effects on weight change over 12 weeks [53]. Therefore, potential differences in energy expenditure and carbohydrate after different non-nutritive sweeteners (e.g., sugar alcohols, saccharin, aspartame, acesulfame K, etc.) ingestion should be investigated in the future.

Observations in our study on appetite ratings and food intake for 24 h, albeit interesting, were not able to draw a conclusion due to the lack of statistical power (this study was powered based on primary outcomes). Our study was not powered to detect differences between dietary intakes after test foods originally due to the absence of other similar human studies. When calculated retrospectively using our own results, at least 117 (Glass’ delta, effect size = 0. 26) participants were needed to detect statistically significant differences in energy intake at α = 0.05 at 80% power, which was beyond the exploratory nature of this study.

## 5. Conclusions

This pilot study provides early findings that the replacement of sugars with NNS during food product formulation, with or without accompanying carbohydrate from maltodextrin, may have implications on human energy balance, especially human energy expenditure and carbohydrate oxidation patterns. Acutely, humans appear to compensate for energy shortfall from NNS but did not promote appetite or overeating beyond that. Findings from this pilot study should be verified and confirmed with bigger clinical trials in the future.

## Figures and Tables

**Table 1:** Energy expenditure, carbohydrate and fat oxidation of 11 participants over 90 min during the indirect calorimetry measurement on SUCROSE, NNS and MALT test days.

	Unadjusted (*n* = 11)				Adjusted (*n* = 11)			
	SUCROSE	MALT	NNS	*p* ^5^	SUCROSE^1^	MALT^1^	NNS^1^	*p* ^5^
Energy expenditure (kJ/90 min)^2^	413 ± 105 ^a^	412 ± 126 ^a^	374 ± 93 ^a^	0.046 *	412 ± 56	410 ± 62	373 ± 55	0.050
Carbohydrate oxidation (kJ/90 min)^3^	311 ± 86 ^a^	256 ± 76 ^b^	191 ± 68 ^c^	0.004 *	311 ± 74 ^a^	255 ± 49 ^b^	191 ± 49 ^c^	<0.001 *
Fat oxidation (kJ/90 min)^4^	104 ± 66 ^a^	157 ± 62 ^b^	159 ± 67 ^a,b^	0.016 *	104 ± 50 ^a^	157 ± 41 ^b^	160 ± 72 ^a,b^	0.015 *

Values are total energy expenditure, carbohydrate oxidation and fat oxidation during the 90 min measurements using the indirect calorimeter. Values are expressed as means ± standard deviations of *n* = 11 participants. SUCROSE – test food with 50g sucrose (sugar), MALT – test food with 120mg sucralose and 50g maltodextrin, NNS – test food with 120mg sucralose only. ^1^ Values were adjusted for body weight measured on test days. ^2^ Unadjusted energy expenditure was significantly lower in NNS test food compared to SUCROSE and MALT test foods (*p* < 0.05). ^3^ Both adjusted and unadjusted carbohydrate oxidation were significantly different between all test foods (SUCROSE > MALT > NNS, *p* < 0.05). ^4^ Both adjusted and unadjusted fat oxidation was significantly lower in the SUCROSE test food than the MALT and NNS test foods (*p* < 0.05). ^5^ *p* values for general linear models for repeated measures ANOVA (GLM RMANOVA). Post-hoc analysis with Bonferroni corrections was performed when overall *p* < 0.05 (*). Values among three test foods with different superscript letters were significantly different (*p* < 0.05).

**Table 2:** Hunger, fullness and desire-to-eat ratings before and after test food ingestion, as well as before and after meal challenge (*n* = 11).

	SUCROSE	MALT	NNS	Time *p*	Test Foods *p*	Time X Test Foods *p*
Hunger (mm)^ 1^				<0.001 *	0.397	0.838
Before jelly 09:00 am	63.3 ± 25.6	72.7 ± 17.7	62.3 ± 21.7			
After jelly 09:10 am	37.7 ± 24.3	48.4 ± 27.8	33.3 ± 21.5			
Pre-meal challenge 11:00 am	63.8 ± 20.6	64.6 ± 15.3	58.0 ± 17.7			
Post-meal challenge 11:30 am	11.8 ± 17.8	15.7 ± 16.3	15.1 ± 19.6			
Fullness (mm)^ 1^				<0.001 *	0.111	0.275
Before jelly 09:00 am	25.9 ± 27.9	16.2 ± 20.9	19.4 ± 19.7			
After jelly 09:10 am	44.8 ± 24.8	23.3 ± 41.0	54.0 ± 19.3			
Pre-meal challenge 11:00 am	27.1 ± 22.3	21.8 ± 15.3	28.0 ± 25.6			
Post-meal challenge 11:30 am	85.5 ± 5.9	78.0 ± 15.2	78.6 ± 9.7			
Desire-to-eat (mm)^ 1^				<0.001 *	0.806	0.656
Before jelly 09:00 am	59.0 ± 32.6	74.7 ± 11.9	64.5 ± 16.6			
After jelly 09:10 am	41.3 ± 26.8	38.4 ± 47.2	40.3 ± 19.2			
Pre-meal challenge 11:00 am	66.0 ± 22.2	69.1 ± 12.5	59.1 ± 22.6			
Post-meal challenge 11:30 am	16.4 ± 21.2	17.5 ± 11.0	19.5 ± 18.4			

^1^ Values are means ± standard deviations of *n* = 11 participants. Time is for reference and based on the assumption that participants started the experiment at 08:00 am on test days, which may vary slightly between participants. * Statistically significant, general linear model for repeated measures ANOVA, *p* < 0.05.
